# Reciprocal Reinforcement Between Wearable Activity Trackers and Social Network Services in Influencing Physical Activity Behaviors

**DOI:** 10.2196/mhealth.5637

**Published:** 2016-07-05

**Authors:** Rebecca Cherng-Shiow Chang, Hsi-Peng Lu, Peishan Yang, Pin Luarn

**Affiliations:** ^1^ School of Management National Taiwan University of Science and Technology Taipei City Taiwan; ^2^ Distinguished Professor at the Department of Information Management School of Management National Taiwan University of Science and Technology Taipei City Taiwan; ^3^ Professor at the Department of Social Work College of Social Sciences National Taiwan University Taipei Taiwan; ^4^ Professor, EMBA Program Director School of Management National Taiwan University of Science and Technology Taipei City Taiwan

**Keywords:** Wearable activity trackers, wearables, physical activity, social support, social network services, behavior change techniques

## Abstract

**Background:**

Wearable activity trackers (WATs) are emerging consumer electronic devices designed to support physical activities (PAs), which are based on successful behavior change techniques focusing on goal-setting and frequent behavioral feedbacks. Despite their utility, data from both recent academic and market research have indicated high attrition rates of WAT users. Concurrently, evidence shows that social support (SS), delivered/obtained via social network services or sites (SNS), could increase adherence and engagement of PA intervention programs. To date, relatively few studies have looked at how WATs and SS may interact and affect PAs.

**Objective:**

The purpose of this study was to explore how these two Internet and mobile technologies, WATs and SNS, could work together to foster sustainable PA behavior changes and habits among middle-aged adults (40-60 years old) in Taiwan.

**Methods:**

We used purposive sampling of Executive MBA Students from National Taiwan University of Science and Technology to participate in our qualitative research. In-depth interviews and focus groups were conducted with a total of 15 participants, including 9 WAT users and 6 nonusers. Analysis of the collected materials was done inductively using the thematic approach with no preset categories. Two authors from different professional backgrounds independently annotated and coded the transcripts, and then discussed and debated until consensus was reached on the final themes.

**Results:**

The thematic analysis revealed six themes: (1) WATs provided more awareness than motivation in PA with goal-setting and progress monitoring, (2) SS, delivered/obtained via SNS, increased users’ adherence and engagement with WATs and vice versa, (3) a broad spectrum of configurations would be needed to deliver WATs with appropriately integrated SS functions, (4) WAT design, style, and appearance mattered even more than those of smartphones, as they are body-worn devices, (5) the user interfaces of WATs left a great deal to be desired, and (6) privacy concerns must be addressed before more mainstream consumers would consider adopting WATs.

**Conclusions:**

Participants perceived WATs as an awareness tool to understand one’s PA level. It is evident from our study that SS, derived from SNS and other pertinent vehicles such as the LINE social messaging application (similar to WhatsApp and WeChat), will increase the engagement and adherence of WAT usage. Combining WATs and SNS enables cost-effective, scalable PA intervention programs with end-to-end services and data analytics capabilities, to elevate WATs from one-size-fits-all consumer electronics to personalized PA assistants.

## Introduction

*Active Aging* (AA) is the public policy framework stipulated by the World Health Organization (WHO) to embrace global aging challenges [1]. A systematic literature review of Social Sciences Citation Index Journal Articles on AA referred to physical activity (PA) as the second most researched determinant in AA, next only to gender [2]. However, the WHO pointed out that physical inactivity levels are still rising in many countries [3]. Wearable activity trackers (WATs) are emerging consumer electronics to assist PA, yet some empirical studies have revealed high attrition rates of WAT users with few solid explanations [4,5]. This study aimed to identify if social support (SS), delivered and obtained via social network services or sites (SNS), can increase WAT users’ engagement and reduce their attrition rate to foster more sustainable PA habits.

### The Importance of Midlife Physical Activity

Research results have demonstrated that there is a direct connection between midlife fitness levels and the onset and frequency of chronic diseases later in life [6]. To promote and maintain health, it is recommended that adults do moderately intensive exercise for at least 30 minutes five days each week, or vigorously intensive activity for at least 20 minutes three days each week [7]. Previous studies in Australia and other countries have revealed that middle-aged adults (45-59 years), particularly men, are least likely to achieve these recommendations [8-10].

### Wearable Activity Trackers’ Gaining Popularity and Looming Problem

WATs are body-worn sensors combined with smart phone apps to allow tracking and recording of one’s PAs [11]. International Data Corporation predicted that the worldwide WAT market will reach a total of 111.1 million units shipped in 2016, and will grow to 214.6 million units in 2019 [12]. PricewaterhouseCoopers’ (PwC) Health Wearables Report pointed out that in 2014, 21% of American consumers already owned wearable devices [13]. Analyses of PA interventions using WATs have shown that goal-setting and frequent behavioral feedback are strongly associated with successful behavior change techniques (BCT) [14-20], by offering objective monitoring rather than relying on self-reports. A systematic review has examined the validity and reliability of WATs by summarizing 22 studies regarding the ability to estimate steps, distance, PA, energy expenditure, and sleep [21]. Available studies have proven the effectiveness of WATs in increasing PA among patients with various health problems [22]. To date, there are few studies probing how WATs may be used in preventive ways (ie, helping people who are still healthy to maintain or increase their PA level). In this study, we intended to focus on middle-aged adults to explore how WATs may help them establish PA behaviors and habits. Although the use of the technology is often considered to be driven by younger age groups, a previous study has indicated that the uptake of wearable devices is bi-modally distributed; younger (25-34 years old) groups use them for fitness enhancement, while older (55-64 years old) groups use them to improve overall health [23].

Despite the boom in the WAT market, there seems to be a looming problem. A recent study indicated that 75% of WAT users stopped using the device in just four weeks [4]. Endeavour Partners’ research in late 2013 revealed that more than half of the US consumers who have owned a modern activity tracker no longer used it after 18 months, and a third stopped within six months [5]. It appears that the adoption of WATs is short-lived, and self-efficacy may have played a role in PA adherence [24]. One must believe in his or her capabilities in successfully executing one’s necessary course of action to satisfy situational demands. Therefore, how to enhance self-efficacy becomes a crucial issue.

### Social Support

SS has great impacts on health, and both the types and sources of SS influence the effects [25]. SS includes four domains of positive and negative social exchange factors. Positive domains are emotional, instrumental, informational, and companionship. Emotional support is defined as others’ expressions of warmth, sympathy, and caring; instrumental support is defined as others’ provision of services and material aid; informational support is others’ provision of advice and information; and companionship is being available or accessible when needed. Negative exchanges correspond to the lack of the four positive domains described above [26,27].

Prior research indicated that WATs have been adopted by individuals seeking to enhance their personal fitness through increased self-monitoring *as well as* social connections with others using the devices [22]. The researchers wondered whether WATs designed with integrated SS functions would be more effective in changing PA behaviors and habits. Alternatively, WATs might reinforce SS, and then change PA behaviors and habits more effectively.

### Social Network Services

SNSs could facilitate the online provision of social relationships to affect health outcomes. While overcoming barriers of physical distance or geographic isolation, SNS could supplement or replace in-person social networks [28]. Previous research has demonstrated the effectiveness of online health intervention programs by providing SS through participants’ sharing, reading, and responding to each other’s messages. Such programs resulted in decreased participant attrition, and increased adherence, completion rate, and engagement. [29-33]. A systematic SS review was undertaken, including a total of 2040 studies identified from eight databases (eg, Scopus, Medline, ProQuest, EMBASE). Of the 2040 studies, 10 met inclusion criteria with interventions using health social network websites, which involved a total of 113,988 participants. Nine of the 10 included studies reported significant improvements in some aspect of health behavior change or outcomes related to behavior change [34]. Facebook was the most utilized SNS, followed by health-specific SNSs and Twitter.

In Asia, variations of SNS have been well-received. The social messaging application LINE surpassed 400 million users worldwide in 2014. Originally in Japan, it is now being used by 75% of the population in Taiwan [35]. Currently, there is little research published in English periodicals on how LINE could be employed as a SS delivery mechanism to influence PA. A small number of studies examined a prototype that facilitated social interactions among users, such as sending messages, greetings, comments, and setting up challenges, rather than allowing data sharing [36]. LINE can provide the aforementioned functions as well as group chat, in which members of the group can set up shared note pages and albums, and manage member lists easily [37].

We believe that SS from interpersonal interactions can achieve constructive reinforcement for using WATs and add a new dimension for designing the products, services, and eco-system. This qualitative study was designed to identify possible correlates between SS and WATs to pave the way for more evidence-based research in the future.

## Methods

This research is a qualitative study using the triangulated approach of both in-depth interviews and focus groups. Triangulation is the application and combination of several research methods in a study of the same phenomenon, and is often used to facilitate validation of data through cross verification from two or more sources. Triangulation also helps ensure the credibility of qualitative analyses [38]. Group dynamics of focus groups allowed us to get richer interactions in areas in which participants had shared experiences or views. In-depth interviews allowed deeper probing of individual attitudes and emotions without interference or peer pressure from other participants. We aimed to probe participants’ PA, WAT usage, and how WATs influenced their PA habits or behaviors. The study period was from August to September of 2015. All in-depth interviews were conducted in Mandarin at cafés or restaurants of the participants’ choice. The three focus groups were undertaken in school lounges. Although discussion guides were developed (see Multimedia Appendix 1), they were not adhered to rigidly, to allow conversations to flow, and participants or interviewers could leave the room for probing when necessary. All discussions were audiotaped and transcribed, and all participants signed a written consent form.

We conducted the first round of three in-depth interviews (two users versus one nonuser), then used the first round’s findings (from initial coding and analysis) to revise our focus group discussion guides. Two focus groups of WAT users (4 and 2 participants each) and one focus group of nonusers (4 participants) were held (see details in Table 1). We were conscious of the gender imbalance (2 female vs 11 male students), so we conducted two additional in-depth interviews with female students (one user vs one nonuser). During these interviews, similar themes were reiterated, and considering time and budget constraints, we deemed that the data had reached satisfactory saturation for the purpose of this study.

### Sampling and Recruitment

We followed the definition of the United Nations on middle-aged adults comprising the group ranging from 40-59 years old [39]. We used purposive sampling to recruit Executive MBA students from National Taiwan University of Science and Technology through the school’s student directory. These executives were middle-aged and tended to lead very busy lifestyles, and found regular PA difficult to attain or maintain. We recruited a total of 15 participants, including 9 WAT users (2 females, 7 males) and 6 nonusers (2 females, 4 males) with a median age of 45. We chose to include both the users and nonusers in our study because we wanted to compare and contrast the PA behaviors and habits between the two groups. Nonusers were potentially former WAT users, and we hoped to gain insights on why they quit using WATs. In this study, nonusers were defined as those who were not currently using WATs.

### Data Collection

All focus groups and in-depth interviews were conducted by the lead author and digitally recorded. The duration of each meeting was approximately 60 to 90 minutes. At the start of each session, the moderator introduced the purpose of the research and the definition of PA based on WHO guidelines [3]. The discussion guide for users was devised to explore four key questions: (1) *what types of WATs are you currently using?,* (2) *for what PA do you use WATs?*, (3) *how does your WAT affect your PA behaviors or habits?*, and (4) *what do you see as the benefits and drawbacks of WATs?*

We started out with open-ended questions with no presumptions. By the end of the first few sessions, a recurring theme of SS emerged throughout the conversation, so we decided to more deeply probe the aspect of SS in ensuing discussions.

### Data Analysis

Trained research assistants transcribed verbatim from the digital audio recordings of each focus group and interview. We applied an iterative and thematic approach to the data by using constant comparative methods and manual coding with no preset categories. Two study authors independently annotated and coded the transcripts, with both identifying emerging topics for discussion as data collection proceeded, and each researcher modified and added codes in light of fresh transcripts. Observations, interpretations, and coded results were periodically compared and debated. Early common themes emerged across the WAT user and nonuser groups, such as the motivations and barriers in carrying out their PA routinely, the importance of SS sources and types of SS, and the SNS that participants had been using to re-enforce their PA behaviors or habits. After the first few sessions’ data were collected and analyzed, SS was used as an overarching analytical framework. The SS lens gave us insights that went much deeper than WATs simply being used as self-monitoring devices.

**Table 1:** Data pertaining to focus groups and in-depth interviews.

Focus groups						
	**Code**	**Code Denotation**	**Age**	**Occupation**	**Function**	**WAT brand, if using**
*Focus group 1 -* *Aug. 26, 2015*						
Nonuser	NFW1	Nonuser, Focus group, Woman, #	48 years old	Telecommunications	Self-Employed Consultant	NA
Nonuser	NFM1	Nonuser, Focus group, Man, #	40 years old	Information Technology	Management Information Systems	NA
Nonuser	NFM2	Nonuser, Focus group, Man, #	44 years old	Health care & Pharmaceutical	Marketing	NA
Nonuser	NFM3	Nonuser, Focus group, Man, #	45 years old	Display Technology	Sales & Business Development	NA
*Focus group 2 -* *Sept. 6, 2015*						
User	UFW1	User, Focus group, Woman, #	43 years old	E-Commerce	Entrepreneur	Xiao-Mi
User	UFM1	User, Focus group, Man, #	55 years old	Building & Construction	Business Development	Xiao-Mi
*Focus group 3 -* *Sept. 12, 2015*						
User	UFM2	User, Focus group, Man, #	40 years old	Information Technology	Management Information Systems	CU (a local brand)
User	UFM3	User, Focus group, Man, #	42 years old	Information Technology	Marketing	Garmin
User	UFM4	User, Focus group, Man, #	54 years old	Information Technology	Technical Support	Xiao-Mi
User	UFM5	User, Focus group, Man, #	45 years old	Media	Administration	Apple Watch, Xiao-Mi
**In-depth interviews**						
	**Code**	**Code Denotation**	**Age**	**Occupation**	**Function**	**WAT brand, if using**
Aug. 19, 2015 User	UIM1	User, In-depth interview, Man, #	51 years old	Education	Administration	Apple Watch
Aug. 20, 2015 User	UIM2	User, In-depth interview, Man, #	50 years old	Telecommunications	Sales & Business Development	Xiao-Mi
Aug. 25, 2015 Nonuser	NIM1	User, In-depth interview, Man, #	45 years old	Financial Services	Sales & Business Development	NA
Sept. 6, 2015 Nonuser	NIW1	User, In-depth interview, Woman, #	50 years old	Semiconductor	Administration	NA
Sept. 19, 2015 User	UIW1	User, In-depth interview, Woman, #	43 years old	Information Technology	Product & Service Development	Apple Watch

## Results

All participants who were WAT users adopted the device to measure their PA in order to improve their health. Underlying the broad umbrella of *health*, there were different individual motivations, including health check-up alarms (UFM4’s diabetes), fear of hereditary fatal diseases (UIM1’s parents both died of cancer), losing weight (UIW1, NIW1), relieving stress (NFM3, UIM1), monitoring sleep quality (UIM2), and desire for looking good (UFW1).

Our study revealed that all participants except for one expressed the need to employ other BCTs and tools such as SS and SNS alongside with WATs to keep themselves motivated. The only user (UIM1) who was satisfied with using WAT alone to assist his PA positioned WAT as a self-monitoring and goal-setting tool. He had been keeping regular exercise and healthy diet for years, which demonstrated a very high level of self-efficacy.

Thematic analysis of this study discovered six themes: (1) WATs provided more *awareness* than *motivation* in PA with goal-setting and progress monitoring, (2) SS, delivered/obtained via SNS, increased users’ adherence and engagement with WATs and vice versa, (3) a broad spectrum of configurations would be needed to deliver WATs with appropriately integrated SS functions, (4) WAT design, style, and appearance mattered even more than those of smartphones, as they are body-worn devices, (5) the user interfaces of WATs left a great deal to be desired, and (6) privacy concerns must be addressed before more mainstream consumers would consider adopting WATs.

The application of the analytical lens of SS helped us focus on what was valued by our participants in their interactions and relationships with their friends, families, peers, and health care and fitness professionals while they were striving to achieve their PA goals.

### Wearable Activity Trackers Provided More Awareness than Motivation in Physical Activity with Goal-Setting and Progress Monitoring

All participants who were WAT users mentioned some triggers for starting to take PA seriously. Responding to the triggers, participants adopted WATs as assistive devices (the means) to help them track their PA progress to achieve their health goals (the end). It was not the WATs *per se* that motivated them to do PA [40].

Both of my parents died of cancer. I am afraid that I have the genes too. My kids are still small and I need to be there for them. So I have been taking PA seriously. I started using pedometers long time ago and have already changed 4-5 trackers since then. Now I am using an Apple Watch. I set the goal to take 10,000 steps a day. After I am done with my work each day, no matter how late it is, I would check to see how far behind I am and make it up by walking or running around the campus.

UIM1

I was diagnosed with diabetes at the age of 52. It scared me to death so I started mountain climbing the very next week. I determined to climb Seven-Star Mountain during weekends as often as possible, rain or shine. Now I have accumulated over 50 climbs in two years (climbed every other week). I used Xiao-Mi to track my steps and distance. Looking back, if I have had not got diabetes, I would not have been so diligent.

UFM4

I bought an Apple Watch because I wanted to lose weight. I am a connoisseur and I am not going to let my weight concerns impede with the biggest hobby of my life.

UIW1

Even with these seemingly strong motivations, most participants still found it difficult to adhere to regular PA. Respondents cited reasons such as being too busy to exercise, finding exercise boring, poor self-management, fear of injury, lack of skills, and lack of encouragement, support, or companionship from family and friends. Simply relying on the device itself was not sufficient.

I started using Xiao-Mi a few months ago. Originally, I set the goal to walk 10,000 steps a day. But, I could hardly adhere to my plan. Speaking of averages, I could only take 3000 to 4000 steps a day. So I gave up.

UIM2

Once I formed the habit of mountain-climbing, I stopped using my Xiao-Mi as it served no purpose any more. Since I climbed by myself, what I really want from my WAT is to have a quick “handshake” function to exchange contact with those familiar faces I bumped into a lot on trails. This would allow us to get to know each other and can help out when necessary, such as in a case of emergency.

UFM4

I bought a Garmin a few years ago, which cost me around 200USD. But I packed it the second day. I realized I could only see the step-counts and my heart-beats. For the rest of the things, such as taking phone calls, checking emails, text messages, etc., I still need to look at my mobile phone. The display screen on the Garmin was too small. It’s redundant to have two mobile devices with me. Moreover, just measuring and recording were not enough for me. I simply do not have time to exercise and the device could only tell me my problems, not solutions.

NIM2

### Social Support, Delivered/Obtained Via Social Network Services or Sites, Increased Users’ Adherence and Engagement with Wearable Activity Trackers and Vice Versa

Based on previous research, sharing daily activity information within a small group of friends was more satisfying and motivating compared to a control group who did not share their information [41]. A small number of existing WAT products do provide such functions to share one’s PA results on Facebook or Twitter, but these systems act as a one-way broadcasting, rather than sharing in a small private group.

Our participants used different social media for different SS. For example, respondents used Facebook to derive emotional and informational SS, and when they achieved PA results on their WATs, they would broadcast about it on Facebook. On bad days when participants fell behind their PA targets, they would keep silent to block possible negative SS. YouTube was also used to get informational SS, such as PA related tips and how-to information.

Although most WATs lack features to support sharing among smaller and more controlled groups, we observed that our participants have devised creative ways to give and receive SS by employing various tools to serve their purposes. For example, LINE social messaging application, which allows conversations within private groups only, has been used by our participants for all four kinds of SS: emotional, informational, instrumental, and even companionship (virtual and physical).

My Executive MBA classmates are all mid to high level managers and supposedly they should be very busy. I joined a jogging club, and we set a common goal to run 5 km per day. I was surprised to find that every evening, I got LINE messages from the club members reporting their progress. If I missed out on a day, they would urge me, “Hey, you owe me one. You’ve got to make it up for me tomorrow!” It’s like in the Army, it’s much easier to run distance together with such peer pressure and team spirit. We also shared tips for warm-up and stretches before and after jogging to prevent from injury. (Emotional, informational and instrumental)

UFM3

I used to exercise regularly when I was young. But over time, I slacked off as my work got busier. What got me back on track was the power of teams. When I started at the Executive MBA program, I joined the biking club and became a team lead. We did island-wide biking trips, which I could not have done by myself. We bought the same brand and model of WATs (Garmin devices) together to track our progress and for the Global Positioning System (GPS) function. We hired a “baby-sitting van” for those trips, which provided drinks, snacks, first-aids, and a coach. It’s a safety net any team member could fall back on if he or she did not feel well. We even put on the uniforms to show our team spirit. (Emotional, instrumental, and companionship SS)

UFM1

For me, I have not used a WAT yet because I didn’t think it would be useful for me. My main problem about not doing PA regularly is because my working hours are long and I simply couldn’t find the time to do it. But after hearing what was being said here in this group, I can relate to the importance of receiving care and reminders from friends and families. Because knowing that someone else cares about my health may give me the strength in overcoming my inertia.

NFM2

UIW1 invited her busy friend to jog with her virtually via LINE.

Since it took too much time to fight the traffic across the town, we decided to jog on the two sides of the same river (we live at the opposite sides) at the same time and to “keep each other posted” along the way through exchanging pictures of our WAT results and beautiful scenery via the LINE messenger app to keep us motivated. We had so much fun “together”! (Emotional and virtual companionship SS).

UIW1

It is not only that SS increased the engagement of WAT usage, but WATs in turn could reinforce SS as well by offering objective progress towards common goals of PA among friends, family, and social groups.

I like my Apple Watch. One day I reached 200% of my goal from jogging. I shared the result on FB and got way more “likes” than usual. I felt good. (Emotional SS)

UIW1

UFM2’s children did not like walking before and got tired easily, so he ended up carrying them home. After UFM2 started using a WAT, it became a virtual “umbilical cord” that bonded them when they exercised together.

My kids never asked me to carry them again as they got excited by looking at their step counts on my WAT and persisted on achieving the daily goal. (Emotional and companionship SS)

UFM2

This finding echoes prior research that indicates wearable devices could be important ways to extend the social networks of physically active people [22]. SS and WATs may be reciprocal, as they reinforce each other while influencing people’s PA behaviors and habits.

### A Broad Spectrum of Configurations Would Be Needed to Deliver Wearable Activity Trackers with Appropriately Integrated Social Support Functions

When considering SS, each person’s needs and sources are different. Our participants cited their SS sources from their family, friends, co-workers, and service providers, such as fitness center coaches and health care professionals.

#### Inter-Generational Social Support is a Major Motivation for Adopting Wearable Activity Trackers

Many participants in this study had children and aging parents, and needed to care for both generations. Inter-generational SS turned out to be a major motivation for adopting WATs. Some participants (UFM2, UFM4, UFW1, and UIW1) intended to buy WATs for their parents to help monitor PA and chronic diseases. Both UFW1’s and UFM3’s fathers had Alzheimer’s disease and the respondents wanted to give them WATs with GPS to track if their fathers had wandered off.

I have told my dad not to ride a motorcycle again. But sometimes, he still “sneaked off” and we would be frantically looking for him. That’s why I want to have him wear a WAT with GPS so that I can track him and find him. (Instrumental SS)

UFW1

UFM4’s and UIW1’s fathers both had diabetes, and they wished to give their fathers WATs to detect falling or to monitor glucose levels.

My dad has been diagnosed with diabetes for many years. We need to monitor his conditions carefully. When the glucose level rose too high, he might faint or fall. It could be dangerous. So I want to give him a WAT to help. It could also help me monitor if he has done PA regularly. If not, I could call to care about him and gently remind him. Or if I have time, I could just go there to accompany him in taking a walk. Otherwise every time I called, he would just tell me that everything was fine. (Emotional, instrumental, and companionship SS)

UFM4

Common barriers mentioned by the participants included statements that their parents were not willing to utilize WATs because they did not like to be “watched” (UFW1) or found WATs cumbersome or hard to use.

Both of my parents live in rural Taiwan. We took it for granted that WAT was already very easy to use. But they didn’t even know how to download an app! (Lacking instrumental support)

UFM3

Some participants intended for their children to wear WATs to help them build endurance through PA (UFM1, UFW1). These participants believed that this tactic would prevent their children from becoming obese (NFM1), or even monitor their sleep quality and sufficiency (UFW1).

I would consider buying WATs for my kids to help them form PA habits, which could build tenacity and endurance to face difficulties in their lives, careers, and future.

UFW1

#### Organizational Social Support from Team Challenges Could Be Transformational in Changing People’s Physical Activity Behaviors and Habits, but Most of the Current Wearable Activity Tracker Designs Lack Team Support Features

Many of the Executive MBA students alluded to their team PA experiences as transformational or even life-changing. Team-building programs offered at schools or companies, such as Charity Marathons (42km or 21km), Mount Jade Climbing (the highest mountain in Taiwan), and Swim Across Sun Moon Lake (the largest freshwater lake in Taiwan) were just a few examples. Such team challenges aroused team spirit, reframed stressful events into developmental opportunities, and inspired members to undertake group missions in order to develop their professional and personal skills [42]. Previous research also suggested that working in a team increased participants’ overall compliance and engagement with electronic health (eHealth) app-based intervention than those assigned to the *solo* condition [32].

WATs have been used as essential tools in team exercises, and the use of WATs could contribute to solidarity and accountability among team members. In order to accomplish the group’s mission, each team member had to sign up for regular practice. Hence, participants needed objective monitoring tools to share their practice results with the team. Nevertheless, many team support features were still lacking in WATs, such as built-in real time communication (eg, walkie-talkie features), GPS locations of each team members (not just one’s own), and emergency alert buttons.

The current WATs lack team support features. Our bike team members had to juggle multiple devices (mobile phone, WAT, GPS, and walkie-talkie, etc.).

UIM1

Once I went on a biking trip with a team, though each one of us had a GPS device, I could only see my own position. I was leading yet I had no idea how far behind my team members were. Even with walkie-talkies, sometimes they fell out of range and we lost contact! It’s quite nerve-wracking! I wish my Garmin could have shown all team members’ locations, as well as the baby-sitting van, real time. I hope my WAT could have a feature of summarizing daily statistics/results of each team member too so that we could adjust our pace and paths for the next day.

UFM1

When we went mountain-climbing in a team, we wish our WATs could tell us if a team member’s oxygen level fell below the safety threshold and immediately triggered an alarm.

UFM4

I am the head of HR and administration and have been in charge of promoting health at my workplace. This year, I convinced my boss to invest in giving each employee a Xiao-Mi bracelet. They used it to form teams to set group goals, to monitor progress, and to join the grand contest hosted by the company. My boss regarded it as a worthwhile investment.

UFM5

#### Social Support from Professional Services Complement Wearable Activity Trackers in Sustaining Physical Activity Behaviors and Habits to Maintain One’s Health

Outside of relying on SS from one’s family, friends, communities, or companies, it is evident that professional service providers such as health care professionals and fitness center coaches play critical roles in providing emotional, informational, instrumental, and companionship SS. Accenture’s 2014 State of the Internet of Things Study found that more than half of consumers were willing to share their wearable data with physicians [38]. WAT producers could design their offers to include such complimentary services to help their customers’ PA results to be more fruitful.

I joined a weight loss program at a municipal hospital, whose health care professionals assessed my health condition, made personal PA advice and kept monitoring my progress toward my goal. I made it! (Informational SS)

UIW1

I loved jogging but started to feel knee pain and stopped. After I consulted a doctor and learned that my knees were fine but the problem lied on my weak muscle due to lack of toning. The doctor suggested that I practice kicking 100 times a day for a few months. Then I resumed jogging and the pain was gone. (Informational SS)

UFM2

I joined a community gym and learned that my moderate walking in parks were not enough. I started working out at the gym. I felt cared for when I got reminder LINE messages to practice from my coach. And I was so touched by them when my fitness center waived my monthly fee for the time I was gone on business trips without my asking for it. (Emotional, informational, and instrumental SS)

NIM1

### Wearable Activity Tracker Design, Style, and Appearance Mattered Even More than Those of Smartphones, as They Are Body-Worn Devices

One female focus group participant, who was not a user, noted that all the models of WAT in the market looked too *macho and sporty* and would not go well with her outfits at work (NFW1). One user expressed his frustration when he wore his US $300 Garmin to work the first time, it was mistaken by a coworker as a toy watch. He was quite vehement about it (UFM3). Two others (one WAT user, one nonuser) said that they definitely would not substitute their brand name watches with WAT bracelets.

There’s no way I am going to trade my IWC (a high-end mechanical Swiss watch) with Apple Watch, not even the Platinum Edition. To me, IWC is part of my identity and personality. When people saw my watch and asked about it, I could proudly tell them its brand story from World War II. Apple Watch has no legacy.

UIM2

I think a WAT bracelet would interfere with my style. I want a Rolex on my wrist. It’s a status symbol. But this? Uh uh.

NFM1

### The User Interfaces of Wearable Activity Trackers Left a Great Deal to be Desired

WAT reminders and prompts aroused reactions ranging from welcoming to annoying. This issue may bear many implications for human computer interface (HCI) design. Persuasive computing could help in this regard, for WAT producers to contemplate further. The concept of persuasive computing was raised by B.J. Fogg in his seminal book, *Persuasive Technology: Using Computers to Change What We Think and Do* [43]. A persuasive system aims to encourage its users to perform speciﬁc actions or tasks, and is based on multidisciplinary ﬁelds involving human-computer interaction and psychology [44,45]. Certain WATs allowed users to set a reminder to stretch periodically. Some participants found it useful, while others felt, “annoyed”, “bothered”, or said, “I would simply ignore it”.

Well, I would not feel “embarrassed” or “ashamed” towards a machine when I am not keeping up with my work-out schedule, but I would with a real human being. So a machine will only work for me if someday, somehow, I would feel “ashamed” toward a machine as well. Ha ha!

NIM1

I don’t like getting “prompts” from a machine to tell me what to do all the time. It’s like my wife’s nagging me, “Hey, you are getting fat! Go out and get some exercise!”

UIM2

### Privacy Concerns Must be Addressed Before More Mainstream Consumers Would Consider Adopting Wearable Activity Trackers

A number of factors were cited by nonusers as causes for their hesitation in adopting WATs. These deterrents included unappealing design (NFW1), uncertainty about their accuracy (NFW1, NFM2, NIM1), lack of user-friendly interfaces (NFM3, UFW1), small and hard-to-read displays (NIM1, NIW1), juggling with multiple mobile devices (NFM2, NIM1), redundancy with existing smartphone fitness apps (NFM2), and high price points (NIM1). However, privacy appeared to be a major concern for nonusers and users alike. This finding is consistent with previous research and market reports. Accenture’s survey indicated that 80% of consumers expressed privacy concerns about personal data-sharing [46]. PwC showed that just one in four respondents said that they wanted to share wearable-device-generated exercise information, or health information in general, with friends and family through social media [13]. This issue could explain why some of our participants expressed hesitation when they thought of sharing their PA progress on SNS such as Facebook. These concerns include minor ones such as losing face, to major ones like fearing to reveal one’s whereabouts unknowingly.

What if I could not reach my weight-loss goal? I would feel embarrassed.

NIW1

Sometimes, I saw on Facebook my friends’ posts such as “check-in at this-and-that airport” or “I ran for 4km at certain park today”. I always wonder why people would want to do that. We are not celebrities. They could have uploaded data to the public accidentally as it’s easy to forget to change the setting from “public” to “friends” only.

NFM3

My Xiao-Mi has built-in buttons to share PA results with my friends via WeChat, Twitter, and even Facebook… Maybe others would. But I won’t. I think LINE is a great invention since it allows us to share things within our private groups instead of broadcasting them on Facebook.

UIM2

## Discussion

### Principal Findings

Current literature reveals that WATs and SNS could be effective in increasing patients’ PA [22]. However, the combined approach of reviewing how SNS and WAT could influence PA behaviors in middle-aged adults together could shed new light in this area of study. Our research demonstrated that these two forces not only work together, but are reciprocal in influencing PA.

Academic and marketing reports regarding WATs have both alerted that the merits of self-monitoring and goal-setting may not be sufficient for most users to form sustainable behavior changes [4,5]. This problem warranted more research to explore how to make the effects of WATs on PA more sustainable.

We began with the working hypothesis that there were gaps in the current designs of WATs. Our study confirmed that SS, delivered/obtained via SNS, increased users’ adherence and engagement with WATs, which in turn reinforced SS in shaping PA behaviors and habits. While most existing research focused on WATs as devices, our study showed that WAT integration with SS services could be indispensable.

Our participants cared much more about WAT design, style, and appearance than about their smartphones', since WATs are body-worn devices. WATs were perceived as part of their personal identities. This finding holds potential for future research to further explore WATs from design and aesthetic perspectives.

More thoughtful HCI designs could be key considerations for adoption as well. Persuasive computing has not been widely applied in WAT designs, which could enrich interactions not just between human and machines, but also among the users’ community, both online and offline [43].

In light of the recent security breaches in all major mobile phone operating systems, and the recent battle between the Federal Bureau of Investigation and Apple regarding encryption, privacy is poised to be a major issue that must be addressed with technological advancements in mobile and wearable devices, and the Internet of things. Consumers’ grave concerns regarding privacy echoed previous research and market reports, so we chose to further elaborate on this theme in the following section regarding implications for practitioners.

### Implications for Practitioners

At the *device* level, we recommend that WAT designs should incorporate built-in group chat functions that allow users to receive and give SS within private, controlled groups. Integrated mutual team support features are highly desirable as well, such as opt-in team members’ GPS locations and emergency alerts.

At the *service* level, participants expressed the need to not just have the measurements or *diagnosis*, but the *personalized prescription* for their PA at the same time. Services should be part of the holistic design from the very beginning, rather than an afterthought. WAT producers may join forces with health care services or fitness centers to position WAT as part of a comprehensive offering to cater to users’ individual health needs.

Finally, at the *policy* level, comprehensive privacy policies need to be in place in order to render WATs as effective public health tools. With GPS and mobile technology becoming so pervasive, while still lacking end-to-end security protection, users may reveal their location unintentionally. It is important for cross-sectorial collaboration to alleviate these concerns, and not simply give these technologies an on-and-off button. Privacy settings need to be designed at a much more granular level (ie, allowing users to select settings in terms of the level, the amount, and the type of data they intend to share, and with whom), and make users aware of who has the right to access and view their personal data. Service providers need to work with authorities and related players in the service eco-system to enhance privacy protection. Government agencies need to work with telecommunication companies and WAT makers to define and regulate the classification of data being transmitted, exchanged, and utilized over public and private networks. Mechanisms must be in place to protect users from being exploited or discriminated against by parties that could potentially threaten or hurt them.

### Limitations

We designed our current research by focusing on the Executive MBA students at National Taiwan University of Science and Technology with participants’ median age at 45, which fit into the United Nation’s *middle-age* category. A digital divide, defined as the economic and social inequality to access and use information technologies, has been a limiting factor in adopting eHealth technologies in older adult cohorts [47]. However, since most of our participants were executives, they did not seem to have such digital divide problems due to their high socio-economic status. Inevitably, such sampling criteria may be biased, and observations derived from this research may not be generalizable to other market demographics. Further studies to probe similarities and differences among various demographics, even psychographic characteristics, would be desirable, such as income levels, professions, genders, personalities, and lifestyles.

Female representation in this study was lower than that of the general population in Taiwan (4 of 15 participants). 2014 marked the first year in history in which there were more women in Taiwan than men, with the ratio being 100 to 99.99 [48]. However, at the management level in Taiwan, females only account for 20% of the workforce [49]. Transferability of findings from this study is therefore limited.

### Suggestions for Future Research

This qualitative research could not explain the trajectory of WAT attrition for a larger user base. However, the discovery that the influences between WAT and SNS are reciprocal is worth further investigation. In addition, research, guidelines, and theories about the adoption of WATs need to keep pace with technological developments. The adoption of WATs integrated with fitness or health care services could enable continuous streams of real users’ data (eg, PA information and bio-signs), which may benefit preventive health research and many medical fields in the future. A pressing need exists for a better understanding of the complexities emerging in the evolution of WATs in the context of real people’s everyday lives and their intertwining social networks, both online and offline. Above all, more research is needed to elevate the standards for privacy protection.

### Conclusion

Our study aimed to identify possible reasons behind the high attrition rate of an emerging category of WATs and to explore possible solutions to make their effects in changing PA behaviors more sustainable. SS, delivered/obtained via SNS or other forms of online community, has been proven to increase the adherence and engagement of PA intervention programs by overcoming barriers and increasing motivations in achieving one’s PA goals.

Our study confirmed that combining WATs and SNS in PA intervention programs with end-to-end services and data analytics could elevate WATs from one-size-fits-all consumer electronic devices to personalized PA assistants to cater to one’s particular health needs. This study also lays the groundwork for more evidence-based research in the future, to realize new possibilities in preventive health enabled by technological advancements.

## Appendix

Multimedia Appendix 1

## Abbreviations

AA: Active Aging
BCT: behavior change techniques
eHealth: electronic health
GPS: Global Positioning System
HCI: human computer interface
PA: physical activity
PwC: PricewaterhouseCoopers
SNS: social network services or sites
SS: social support
WAT: wearable activity trackers
WHO: World Health Organization
