# Supplementary material for: Reciprocal Reinforcement Between Wearable Activity Trackers and Social Network Services in Influencing Physical Activity Behaviors
Source: JMIR Mhealth Uhealth. 2016 Jul 5;4(3):e84. doi: 10.2196/mhealth.5637 (PMC4951629; doi:10.2196/mhealth.5637)
Supplement: Multimedia Appendix 1 [file mhealth_v4i3e84_app1.pdf]

Multimedia Appendix 1

Discussion Guide for focus groups and in-depth interviews.

Preparations were the same for both WATs' users and non-users:

1. Introduce the format of the focus group/in-depth interview.
2. Remind them to just share their personal experiences and there are no right or wrong answers.
3. Show them the definition of PA by WHO.
4. Show them the definition of WATs based on previous research.

Discussion Guide for WATs' users:

I. How would you evaluate your current health condition? Are you happy with it?

II. What physical activities are you doing? Please describe your frequency and intensity level. Do you do it by yourself or with other people? If the latter, whom are you doing it with? Are you happy with what you are doing now? Why and why not?

III. What triggered you to start using wearable device trackers? How long have you been using it? What physical activities do you use it for? To measure what? How would you describe your experiences so far? Has it changed your physical activities habits in any way and how? What features do you use most often and least often? What functions do you like the most and the least? What features do you wish your device could offer? Why would you want those features, and how are you going to use them?

IV. Are there any other family members or friends that are using wearable device trackers? How would they describe the experiences? Would you recommend them to use WATs if they have not, and what would you suggest that they use WATs for? Are you or would you be using WATs to do anything together? Please describe.

V. There are a few common features related to physical activities that we have seen in WATs in the market, including the ones you are using, such as the below. Among all the features listed, what do you think of them? Which ones appeal to you the most and the least? Why?

   A. Goal setting
   B. Progress measurement
   C. Feedback: encouragement, reminder, etc.
   D. Advice and tips: e.g. how to do a warmup?
   E. Detection of the sport you are undertaking (walking, biking, etc.) to provide the right kind of measurement, such as distance, steps, time, etc.
   F. GPS for trail records or navigation

G. Mood detection

H. Sharing on SNS

I. Show simulated consequences of your choice: if you do not exercise, what could happen (show a picture with you becoming fat)

J. Reinforcement: link your physical activities with other good deeds. For example, if you exercise this much, we will plant a tree for you, etc.

Discussion Guide for non-users:

I. How would you evaluate your current health condition? Are you happy with it?

II. What physical activities are you doing? Please describe your frequency and intensity level. Do you do it by yourself or with other people? If the latter, whom are you doing it with? Are you happy with what you are doing now? Why and why not?

III. What do you think of wearable device trackers? Have you considered using one? Why and why not?

IV. Are there any other family members or friends that are using wearable device trackers? How would they describe their experiences?

V. There are a few common features we have seen in the WATs in the market, such as the below. Among all the features listed, what do you think of them? Which ones would appeal to you the most and the least? Why?

A. Goal setting

B. Progress measurement

C. Feedback: encouragement, reminder, etc.

D. Advice and tips: e.g. how to do a warmup?

E. Detection of the sport you are undertaking (walking, biking, etc.) to provide the right kind of measurement, such as distance, steps, time, etc.

F. GPS for trail records or navigation

G. Mood detection

H. Sharing on SNS

I. Show simulated consequences of your choice: if you do not exercise, what could happen (show a picture with you becoming fat)

J. Reinforcement: link your physical activities with other good deeds. For example, if you exercise this much, we will plant a tree for you, etc.
